# Rolling uphill: in vivo reacquisition of pluripotency during cranial neural crest differentiation

**DOI:** 10.1038/s42003-021-02154-6

**Published:** 2021-05-20

**Authors:** Fanju W. Meng, Patrick J. Murphy

**Affiliations:** Department of Biomedical Genetics, University of Rochester Medical Center, Rochester, NY USA

## Abstract

Conrad Waddington famously used his epigenetic landscape to describe the paths a cell might take during developmental differentiation. In this analogy, the undifferentiated stem cell begins at the highest elevation and proceeds to tumble downward towards its final resting place, representing terminal differentiation. This general concept elegantly captures the essence of developmental transitions, but recent single-cell studies by Dr. Joanna Wysocka’s research group indicate that an alternative strategy underlies development of cranial neural crest cells. Published in *Science*, Antoine Zalc, Rahul Sinha and colleagues discovered that ectoderm-derived cranial neural crest cells undergo a developmental reprogramming event in vivo, expanding their differentiation potential through the reactivation of pluripotency, in effect rolling backwards up Waddington’s development landscape before eventually differentiating into mesenchymal lineages.


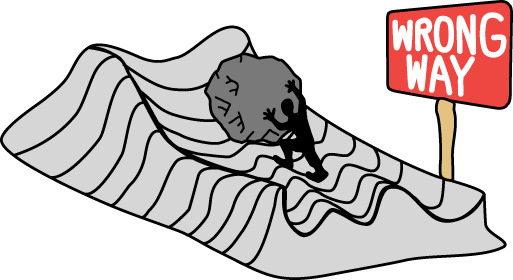


Cranial neural crest cells (CNCCs) originate from ectoderm but exhibit remarkable multipotent differentiation potentials, deriving a variety of cell types, including the cartilage and bone in the craniofacial skeleton, which are usually derived from the mesoderm. The molecular mechanism by which CNCC differentiation potential expands beyond the three germ layers remains largely unknown.

A recent study from Joanna Wysocka’s research group, led by Antoine Zalc and Rahul Sinha at Stanford University, suggests that murine CNCCs acquire differential potential of ectomesenchyme through transient reactivation of the pluripotency program^1^. Using single cell transcriptomics of murine developing CNCCs from embryonic day (E) 8 to E8.75, authors discovered that the pluripotency factors such as *Sox2*, *Oct4* and *Nanog* are expressed in early *Wnt1*-expressing CNCC precursors. By tracking dynamics of *Oct4* expression, they showed that early-developing neural folds do not express *Oct4*, but *Oct4* is reactivated in the prospective CNCC domain, at the onset of somitogenesis. They then performed lineage tracing and genetic ablation of *Oct4* expressing CNCC precursors, finding that Oct4-positive precursors were essential for proper development of craniofacial structures. Despite the absence of Oct4, neural and glial derivatives were normal, suggesting that Oct4 is dispensable for CNCC induction but required for ectomesenchyme specification and survival. At the level of enhancer chromatin, Oct4-positive CNCC precursors appeared quite similar to pluripotent epiblast stem cells, suggesting that priming of the *cis*-regulatory landscapes precedes the acquisition of mesenchymal potential.

This study provides mechanistic insight for how developmental acquisition of multipotency is achieved in CNCCs, and offers a sophisticated example of development where differentiation is achieved by reassembling a prior cellular program rather than tumbling down a preordained cascade.
